# Uncovering the New Biology of Giant Cell Arteritis to Guide Therapeutic Strategies

**DOI:** 10.3390/jcm14186350

**Published:** 2025-09-09

**Authors:** Mayu Shiomi, Ryu Watanabe, Ryuhei Ishihara, Sayaka Tanaka, Goichi Kageyama, Motomu Hashimoto

**Affiliations:** 1 Department of Rheumatology, Hyogo Prefectural Amagasaki General Medical Center, Amagasaki 660-8550, Japan; 2 Department of Clinical Immunology, Graduate School of Medicine, Osaka Metropolitan University, 1-4-3, Asahi-Machi, Abeno-Ku, Osaka 545-8585, Japan; 3 Department of Legal Medicine, Graduate School of Medicine, Osaka University, Suita 565-0871, Japan

**Keywords:** clonal hematopoiesis, epigenetic alterations, giant cell arteritis, immunosenescence, targeted therapy, multi-omics

## Abstract

Giant cell arteritis (GCA) is a form of large vessel vasculitis that primarily affects older adults and forms granulomatous inflammation in the aorta and its major branches. Recent advances in immunology and multi-omics technologies have elucidated several key mechanisms involved in the pathogenesis of GCA, including immune checkpoint dysregulation, clonal hematopoiesis, and age-associated immune dysfunction. From the perspective of immune cell subsets, a diverse range of immune cells—including tissue-resident memory T cells, stem-like T cells, macrophage subsets, B cells, and myofibroblasts—play distinct roles in sustaining vascular inflammation and tissue remodeling. This review summarizes the latest immunopathological and omics-based insights into GCA, proposes potential therapeutic targets, and discusses future directions for precision medicine aimed at achieving sustained remission.

## 1. Introduction

Giant cell arteritis (GCA) predominantly affects individuals over 50 years of age and is the most common form of vasculitis in the elderly. It primarily involves the aorta and its major branches [1]. Epidemiologically, GCA has the highest prevalence in North America and Northern Europe and is relatively rare among East Asian populations [2].

Clinical manifestations vary depending on the arteries involved. For example, disease affecting the external and internal carotid arteries may present with visual loss, headache, or jaw claudication, whereas vertebral artery involvement can result in stroke. When the subclavian or axillary arteries are affected, patients may develop arm claudication and diminished pulses. While stenosis and occlusion are characteristic of medium-sized arteries, thoracic aortic disease can lead to severe complications, including aneurysm formation, dissection, and even rupture [1]. Early-onset GCA is often characterized by pronounced systemic symptoms and frequent relapses, whereas late-onset GCA is more commonly associated with intracranial involvement and comorbidities [3,4].

In recent years, advances in immunological omics-based analytical techniques have significantly enhanced our understanding of the pathophysiology of GCA. Emerging insights have revealed novel perspectives, including the interplay between aging and inflammation, epigenetic modifications, and the intricate networks of various immune cell subsets and cytokines. In this review, we summarize the latest findings on the emerging pathogenic mechanisms underlying GCA and discuss potential future therapeutic strategies informed by these insights.

## 2. Histopathology

**Figure 1:**
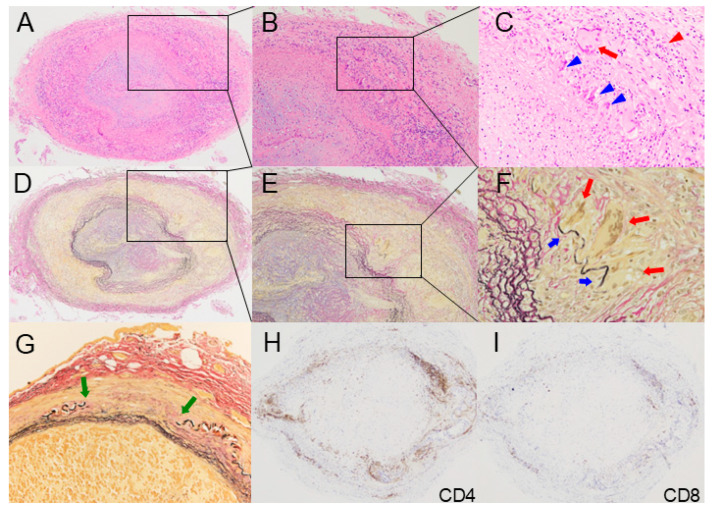
Representative histopathological features of temporal artery specimens from patients with giant cell arteritis. (**A**) Typical giant cell arteritis. Inflammation mainly in the adventitia, extending to the media and the intima. Concentric intimal hyperplasia with luminal occlusion. Hematoxylin and eosin stain (H&E), ×40. (**B**) Higher-power view of A. Prominent inflammatory cell infiltrates in the adventitia, extending into the media, with relatively sparse infiltration in the hyperplastic intima. H&E, ×100. (**C**) Granulomatous inflammation. Macrophages (blue arrowheads) aligned at the media–intima junction with multinucleated giant cell (red arrow). Lymphocyte-predominant inflammatory infiltrates in the adventitia (red arrowhead). H&E, ×200. (**D**) Elastica van Gieson stain (EVG), low magnification, ×40. (**E**) Higher-power view of D. EVG, ×100. (**F**) Infiltration of multinucleated giant cells (red arrows) with phagocytosing elastic fibers (blue arrows). EVG, ×400. (**G**) Disruption of the internal elastic lamina (green arrows). EVG, ×200. (**H**) CD4^+^ T-cell infiltration from the adventitia into the media–intima junction. CD4 stain, ×40. (**I**) Minimal infiltration of CD8+ T cells. ×40.

The histopathological examination of temporal artery biopsy (TAB) remains the gold standard for the definitive diagnosis of GCA. The characteristic finding is concentric thickening of the vascular wall with marked intimal hyperplasia, which contributes to vascular occlusion and subsequent ischemic complications. Granulomatous inflammation is typically observed at the media–intima junction, characterized by prominent infiltration of CD4^+^ T cells and macrophages, often accompanied by multinucleated giant cells. Another key diagnostic feature is the destruction or fragmentation of the internal elastic lamina, which can be clearly demonstrated using elastin stains such as EVG. The inflammatory infiltrate consists predominantly of CD4^+^ T cells and macrophages, with only a minor contribution from CD8^+^ T cells. Additional features include neovascularization and adventitial inflammation, both reflecting ongoing vascular injury and reparative processes. Ultimately, persistent vascular inflammation results in intimal hyperplasia with vascular occlusion, or in arterial wall destruction leading to aneurysm formation (Figure 1) [1].

## 3. Pathophysiology

**Figure 2:**
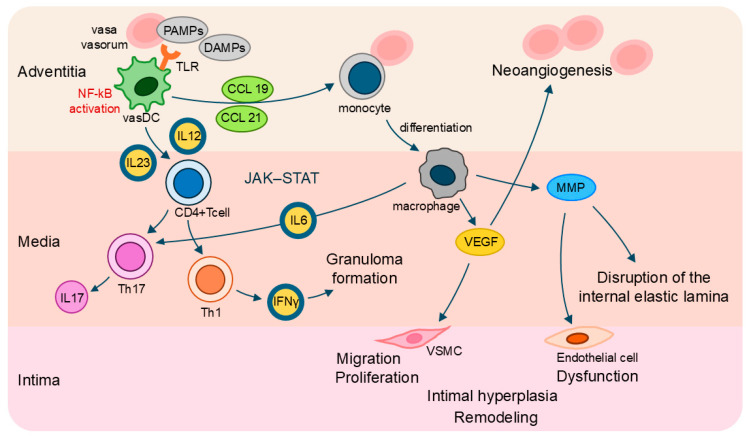
Inflammatory signaling pathways and vessel wall remodeling. PAMPs and DAMPs activate vasDCs in the adventitia through TLRs, leading to NF-κB activation and production of cytokines such as IL-12 and IL-23, as well as chemokines (CCL19, CCL21) that recruit monocytes and CD4+ T cells. Monocytes differentiate into macrophages, which contribute to granuloma formation and tissue damage. Activated vasDCs promote differentiation of naïve CD4+ T cells into Th1 and Th17 subsets via IL-12 and IL-23, respectively. Th1 cells secrete IFNγ and Th17 cells produce IL-17, while IL-6 further amplifies T-cell activation. IL-6, IL-12, IL-23, and IFNγ signal through the JAK–STAT pathway to sustain T-cell activation and chronic inflammation. Macrophages release VEGF, inducing neoangiogenesis, and MMPs, which disrupt the internal elastic lamina. These processes lead to VSMC migration and proliferation, endothelial cell dysfunction, intimal hyperplasia, and vascular remodeling. Abbreviations: CCL, chemokine-C motif ligand; DAMPs, damage-associated molecular patterns; IFN, interferon; IL, interleukin; MMP, matrix metalloproteinase; PAMPs, pathogen-associated molecular patterns; TLR, Toll-like receptor; vasDC, vascular dendritic cells; VEGF, vascular endothelial growth factor; VSMC, vascular smooth muscle cell.

The pathophysiology of GCA involves a complex interplay of genetic predisposition, age-related immune dysfunction, environmental triggers, and aberrant immune signaling. Vascular dendritic cells (vas-DCs) residing at the media–adventitia border are activated upon stimulation through Toll-like receptors (TLRs), leading to the release of IL-12 and IL-23. These cytokines promote the differentiation of recruited CD4+ T cells into Th1 and Th17 subsets. Simultaneously, monocytes differentiate into macrophages that produce interleukin-6 (IL-6), matrix metalloproteinases (MMPs), tumor necrosis factor-α (TNF-α), and vascular endothelial growth factor (VEGF), thereby contributing to vascular wall destruction and remodeling. VEGF not only promotes neovascularization but also contributes to fibroblast proliferation and adventitial fibrosis. Myofibroblasts proliferate within the intima, resulting in intimal hyperplasia [5,6]. A schematic overview of these inflammatory signaling pathways is provided in Figure 2. It is important to further dissect these classical inflammatory cascades not only from the perspective of genetic susceptibility but also in the context of various immune dysregulations and immunosenescence.

### 3.1. Genetic Factors

Genetic susceptibility is an essential factor in understanding the pathophysiology of GCA, and it has long been associated with the HLA-DRB1*04 allele [7,8].

The largest genome-wide association study (GWAS) to date, involving GCA patients from Europe and North America, confirmed previously established associations with HLA class II alleles and the plasminogen gene, and additionally identified three novel genetic loci. These include *MFGE8* and *VTN*, both implicated in angiogenesis, as well as *CCDC25*, a gene associated with neutrophil extracellular traps (NETs) [9]. Although the HLA-DRB1*15 haplotype has previously been considered protective against GCA, recent evidence suggests its involvement in classical cranial GCA, with the HLA-B15:01 allele frequently observed in patients with large-vessel involvement [10]. Most recently, integrative analyses combining single-cell RNA sequencing with Mendelian randomization have suggested that three genes—*RCAN3*, *RPS6*, and *HLA-DQB1*—may confer a protective effect against the development of GCA [11].

Our recent work identified additional genetic risk factors. The GWAS data from the UK Biobank and FinnGen cohorts revealed that the T allele of the SNP *rs7044155*, located within the very low-density lipoprotein receptor gene region, is associated with an increased risk of developing GCA. Furthermore, the same study employed Mendelian randomization analysis, suggesting that elevated levels of low-density lipoprotein cholesterol may contribute to the risk of GCA onset [12].

These advances in genetic research have substantially expanded our understanding of the heritable components of GCA, and ongoing investigation is expected to further elucidate the genetic architecture underlying disease susceptibility.

### 3.2. Environmental Factors

Environmental factors, such as bacterial or viral infections (e.g., Mycoplasma pneumoniae, Chlamydia pneumoniae, Parvovirus B19, Herpes Zoster Virus), have been proposed as triggers for GCA. Although direct invasion of the vascular wall by infectious pathogens and deposition of immune complexes have been proposed as potential mechanisms of vascular injury, no studies have definitively demonstrated these processes, and the supporting evidence remains limited [13,14]. Reductions in beneficial microbes such as Lachnospiraceae and Bacteroidales may disrupt regulatory T-cell (Treg) homeostasis and promote Th17 polarization, thereby contributing to the development of vascular inflammation [15,16].

### 3.3. Immune Checkpoint Dysregulation

While genetic studies have shed light on the heritable predisposition to GCA, accumulating evidence highlights the critical role of immune checkpoint dysregulation in its pathophysiology [5]. In vascular lesions of patients with GCA, vascular dendritic cells exhibit reduced expression of the immunosuppressive ligand programmed death-ligand 1 (PD-L1), whereas infiltrating CD4+ T cells display elevated expression of programmed death-1 (PD-1) [17,18]. Supporting these findings, a study using *C57BL/6* mice demonstrated that, under non-inflammatory conditions, vascular-resident dendritic cells express higher levels of PD-L1 compared to splenic dendritic cells, while PD-L1 expression is significantly downregulated following inflammation induced by lipopolysaccharide [19]. These observations collectively support the concept that local immune checkpoint dysfunction plays a crucial role in GCA.

While immune checkpoint signaling is locally suppressed in vascular tissues, patients with GCA and polymyalgia rheumatica (PMR) exhibit elevated systemic levels of soluble immune checkpoint molecules (sICMs), including soluble cytotoxic T-lymphocyte antigen 4 (sCTLA-4), sPD-1, sPD-L1, and sPD-L2, compared to healthy individuals. These molecules have shown strong discriminatory ability in distinguishing PMR and GCA from healthy controls [20]. Additionally, patients with GCA exhibit significantly increased frequencies of PD-1-positive circulating peripheral helper T cells and T follicular helper cells compared to healthy controls [21]. These findings suggest that systemic immunoregulatory mechanisms may be activated in these conditions. However, the functional relevance of these elevated sICM levels remains unclear, and it is not yet known whether these immunosuppressive pathways are effectively functioning.

The possible association between immune checkpoint inhibitors and large-vessel vasculitis has also been reported. In a human artery-engrafted Severe Combined Immunodeficiency (SCID) mouse model, injection of peripheral blood mononuclear cells (PBMCs) from GCA patients followed by administration of an anti-PD-1 antibody resulted in marked T-cell infiltration into the vessel wall, accompanied by excessive production of proinflammatory cytokines such as IFN-γ, IL-17, and IL-21. Histologically, this was associated with pronounced intimal hyperplasia, neoangiogenesis, and vascular remodeling [17].

Furthermore, clinical studies have documented a significantly increased incidence of GCA in cancer patients treated with the anti-CTLA-4 antibody ipilimumab. In patients with GCA, dysfunctional Treg overexpress CTLA-4, which may increase their sensitivity to CTLA-4 blockade by ipilimumab [22]. These findings suggest that the CTLA-4 pathway plays a critical role in the immunopathogenesis of GCA.

Recent studies have also identified functional impairments in two major inhibitory checkpoint pathways in GCA, the PD-1/PD-L1 axis and the CD96/CD155 axis, leading to the concept of “checkpoint dysfunction” [23]. This weakening of inhibitory signaling is believed to promote persistent T-cell activation and sustained generation of proinflammatory effector T cells, including Th1, Th17, and Th9 subsets [23]. Th1 cells enhance inflammation via IFN-γ production, while Th17 cells stimulate vascular endothelial cells to secrete IL-6 and granulocyte–macrophage colony-stimulating factor (GM-CSF), which in turn activate macrophages and drive vascular remodeling. Additionally, reduced expression of CD155 on antigen-presenting cells (APCs) may impair CD96-mediated T-cell suppression, thereby perpetuating inflammation and aggravating vasculitis through expansion of Th9 cells [23].

These findings underscore the need for further research to clarify the regulatory mechanisms governing immune checkpoint expression in vascular inflammation.

## 4. Aging and Inflammation

GCA predominantly affects individuals over the age of 50 years, suggesting that aging is a significant risk factor for disease onset. Recent advances in aging research have increasingly highlighted physiological changes, as well as the roles of immunosenescence and senescence-associated immune dysregulation, in the pathogenesis of GCA. The concept of inflammaging has recently emerged to describe the phenomenon of chronic, low-grade systemic inflammation that develops with age in the absence of overt infection and has been reported to be associated with persistent elevations of proinflammatory cytokines, including IL-6 and TNF-α [24,25,26,27,28,29]. As central mechanisms of inflammaging, age-related mitochondrial dysfunction and epigenetic alterations are believed to drive cellular senescence, with senescent cells acquiring a senescence-associated secretory phenotype (SASP) that further amplifies chronic inflammation [30].

In hematopoietic stem cells (HSCs), aging is associated with chromatin remodeling that facilitates the transcription of genes involved in inflammation and oxidative stress. This shift promotes the preferential generation of myeloid-biased HSC subsets over lymphoid-biased subsets. Notably, while myeloid-biased HSCs retain their self-renewal capacity, the self-renewal ability of lymphoid-biased HSCs declines with age. These findings may partially explain the diminished immune competence observed in elderly individuals [31]. Recent evidence indicates that clonal hematopoiesis of indeterminate potential (CHIP), an age-associated somatic mutation in hematopoietic stem cells, contributes to sustained inflammation through the emergence of immune cells with heightened pro-inflammatory cytokine production [32].

**Figure 3:**
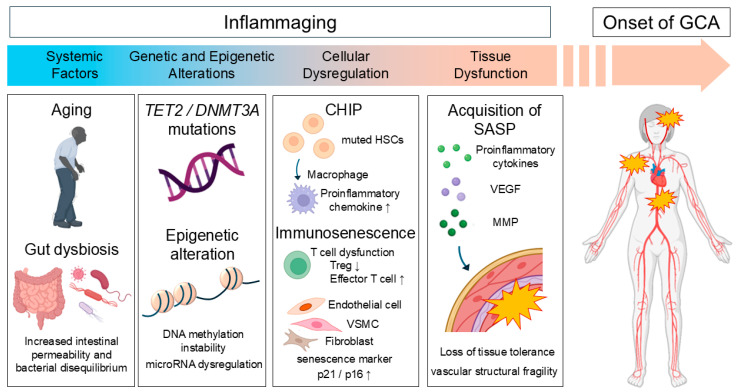
Proposed mechanisms underlying the onset of giant cell arteritis through age-related immune dysregulation and inflammaging. The onset of GCA is driven by a complex interplay of systemic, genetic, epigenetic, and cellular factors, all of which collectively contribute to vascular tissue dysfunction. Systemic aging and gut dysbiosis increase intestinal permeability and disrupt microbial homeostasis, thereby promoting systemic inflammation. Genetic and epigenetic alterations—such as *TET2* and *DNMT3A* mutations, DNA methylation instability, and microRNA dysregulation—further enhance immune activation. CHIP leads to the emergence of proinflammatory macrophages, while immunosenescence impairs T-cell function, Treg populations, and promotes effector T-cell responses. Senescent cells in vascular and stromal compartments—including endothelial cells, VSMC, and fibroblasts—are characterized by elevated expression of p21 and p16. These cells acquire SASP, producing proinflammatory cytokines, VEGF, and MMPs, which compromise tissue tolerance and vascular integrity. The convergence of these pathological processes may ultimately result in the clinical onset of GCA. Abbreviations: CHIP, Clonal hematopoiesis of indeterminate potential; MMPs, matrix metalloproteinases; SASP, Senescence-associated secretory phenotype; Treg, Regulatory T cell; VSMC, vascular smooth muscle cell.

This persistent inflammatory state is believed to contribute to the development of age-related diseases, including atherosclerosis, heart failure, stroke, and vascular stenosis [32,33]. Recent evidence further suggests that inflammaging may contribute to the pathophysiology of GCA [33,34]. In this section, we summarize the key features of these mechanisms and examine the potential impact of age-related immunological changes on GCA (Figure 3).

### 4.1. Epigenetic Alterations

Among the mechanisms linking aging to chronic inflammation in GCA, epigenetic alterations have emerged as key contributors. Genomic instability plays a critical role in the aging process and is accompanied by diverse epigenetic changes, typically involving transcriptional regulation through DNA methylation, histone modifications, and transposable elements. Post-transcriptional gene regulation is further mediated by non-coding RNAs such as microRNAs (miRNAs) [35,36]. Notably, the epigenetic alterations observed in patients with GCA appear to overlap with those found in senescent T cells [37,38].

Epigenetic profiling using genome-wide DNA methylation arrays on TAB specimens from GCA patients revealed hypomethylation events associated with increased activity of the calcineurin/nuclear factor of activated T cells (NFAT) pathway compared to healthy controls [37].

NFATC1, a member of the NFAT transcription factor family, was localized to CD3+, CD4+, and CD8+ T lymphocytes, as well as CD68+ macrophages infiltrating the vascular wall in TAB specimens. Within these tissues, several pro-inflammatory miRNAs associated with T-cell activation, including those regulated by NFATC1, were found to be upregulated. In contrast, multiple regulatory immune-related miRNAs were downregulated. These findings suggest that dysregulation of the calcineurin/NFAT signaling pathway may impair the proper execution of T cell-mediated immune responses [39].

One such miRNA, *miR-21*, is known to increase with age and promote the differentiation of activated CD4+ T cells from memory to inflammatory effector phenotypes, thereby contributing to vascular remodeling [40,41]. Importantly, elevated levels of miR-21 have been demonstrated in inflamed temporal arteries from GCA patients [38]. Although a causal relationship between these epigenetic changes and the development of GCA has not yet been established, these aberrantly expressed miRNAs in TAB specimens from GCA patients may serve as potential diagnostic biomarkers or therapeutic targets.

### 4.2. Clonal Hematopoiesis of Indeterminate Potential (CHIP)

In addition to epigenetic modifications, clonal alterations in HSCs have also been recognized as important age-associated contributors to chronic inflammation. CHIP refers to the age-related acquisition of somatic mutations in HSCs—most commonly loss-of-function mutations in *DNMT3A* and *TET2*—in the absence of overt hematologic malignancy or other known clonal disorders. CHIP is defined by the presence of such somatic mutations in peripheral blood with a variant allele frequency of ≥2% [42,43].

Recent murine studies have demonstrated that aged mice exhibit increased intestinal permeability and microbial dysbiosis, both of which correlate with the expansion of mutant HSCs. Notably, treatment with broad-spectrum antibiotics resulted in a reduction in these mutant HSCs. Conversely, fecal microbiota transplantation from colitic or aged mice into healthy recipients promoted the expansion of *DNMT3A*-deficient HSCs. These findings suggest a potential role for the gut microbiome in driving the clonal expansion of mutant HSCs [44]. In particular, recent work has identified ADP-D-glycero-β-D-manno-heptose (ADP-heptose)—a metabolic byproduct specific to Gram-negative bacteria—as a key microbial-derived ligand. ADP-heptose activates transcriptional reprogramming and NF-κB signaling via the host receptor *ALPK1*, thereby promoting CHIP development [45,46].

Once somatic mutations arise, monocytes derived from these mutant HSCs can be recruited from the bloodstream into atherosclerotic plaques, where they produce excessive amounts of IL-6, IL-1β, and chemokines, contributing to the progression of atherosclerosis [47,48,49,50]. CHIP has also been associated with hematologic malignancies and may influence the efficacy and toxicity of therapies such as hematopoietic stem cell transplantation and CAR-T cell therapy [51]. In solid tumors, *TET2* mutations in CHIP have been linked to tumor-infiltrating clonal hematopoiesis, which in turn has been associated with an increased risk of recurrence and mortality in patients with solid cancers [52,53]. Moreover, CHIP—particularly *DNMT3A* mutations—has been implicated in osteoporosis through IL-20-mediated osteoclast activation [54], as well as in the exacerbation of periodontitis via enhanced osteoclast activity [55].

More recently, CHIP has been reported to be associated not only with autoinflammatory syndromes such as VEXAS syndrome, but also with autoimmune diseases, including rheumatoid arthritis, systemic lupus erythematosus, and vasculitis [32,56,57,58,59]. A study investigating the prevalence of CHIP in patients with GCA, Takayasu arteritis (TAK), and ANCA-associated vasculitis (AAV) found that CHIP was detected in 60% of GCA patients, including in temporal artery tissue specimens. The frequency was higher than that in age-matched healthy individuals and greater than that in patients with TAK or AAV. However, other studies have shown that the difference between GCA patients and healthy controls becomes statistically insignificant after adjusting for age; thus, the true prevalence of CHIP in GCA remains uncertain [58,60,61].

In ex vivo stimulation assays using macrophages derived from GCA patients, those harboring CHIP produced significantly higher levels of inflammatory chemokines, including MCP-1, MIP-1α, and MIP-1β. Moreover, a higher variant allele frequency of mutated clones was associated with an increased risk of relapse [61], particularly among patients carrying *TET2* or *JAK2* mutations [60]. Notably, *TET2* mutations have been linked to an elevated risk of ischemic vision loss in GCA [62,63]. Additionally, male GCA patients exhibited a 4.8-fold increased risk of developing myeloid malignancies such as myeloproliferative neoplasms compared to the general population, with a high prevalence of *JAK2 V617F* mutations among affected individuals [64]. GCA cases harboring *JAK2* mutations were characterized by fewer cranial symptoms, such as headaches, and elevated platelet counts [65].

Interestingly, while CHIP has traditionally been attributed solely to somatic genetic mutations, recent evidence suggests a possible contribution of DNA methylation instability (DMI). DMI refers to increased variability in methylation levels at CpG sites that are normally stable. This phenomenon has been associated with disease progression and relapse in hematologic malignancies, cardiovascular disease, and aging. Although a direct causal link to CHIP has not yet been established, DMI may represent a novel, mutation-independent mechanism contributing to CHIP development [66].

In summary, mutant clones arising from CHIP may contribute to the chronic inflammatory response and risk of relapse in GCA through the excessive production of proinflammatory cytokines and chemokines. However, whether CHIP directly contributes to the onset of GCA remains uncertain, and definitive evidence is currently lacking.

### 4.3. Immunosenescence

Immunosenescence encompasses both the age-related functional decline of immune cells in the innate and adaptive immune systems and cellular senescence, characterized by cell-cycle arrest, evasion of apoptosis, and acquisition of a SASP. This process is recognized as a major driver of inflammaging. For instance, DCs exhibit age-associated impairments in TLR function, increased production of proinflammatory cytokines, and heightened reactivity to self-antigens [67]. In T cells, age-related changes are linked to thymic involution, leading to alterations in both composition and function. Specifically, aging is associated with a reduction in naïve T cells and Tregs, along with a relative expansion of memory and effector T-cell populations [68,69,70,71,72]. In patients with GCA, a decreased number of CD4+ Tregs has been reported compared to healthy controls, suggesting their involvement in disease pathogenesis [73]. More recently, attention has shifted toward functional defects in CD8+ Tregs and their association with autoimmune diseases [68]. In GCA, CD8+ Treg deficiency has been clearly identified. Aberrant signaling via NOTCH4 receptors expressed on CD8+ Tregs disrupts the secretion of exosomes containing NOX2, resulting in a failure to suppress CD4+ T-cell activation and thereby contributing to persistent and exacerbated inflammation [74]. Moreover, dysregulation of glycolytic enzymes and reduced T-cell receptor (TCR)-induced calcium influx have also been implicated in Treg dysfunction in GCA [75].

Interestingly, aging markers such as p21 and p16 are highly expressed not only in macrophages and T cells infiltrating the temporal arteries of GCA patients, but also in vascular smooth muscle cells and fibroblasts [76]. The senescence of vascular endothelial and smooth muscle cells induces a so-called SASP, characterized by the production of inflammatory cytokines (e.g., IL-6, IL-1β), growth factors (e.g., VEGF), and MMPs [77]. This SASP contributes to structural fragility of the vessel wall and breakdown of tissue tolerance, creating a microenvironment conducive to the onset of GCA [78]. This tissue damage is mediated via IL-6-dependent pathways, and tocilizumab has been suggested to suppress this inflammation-promoting mechanism [79].

Taken together, immunosenescence, characterized by the reduction in and functional impairment of Tregs, may permit excessive activation of pro-inflammatory T cells and thereby contribute to vascular wall inflammation. In addition, cellular senescence, through the SASP, may help sustain and amplify the inflammatory milieu. These processes may act synergistically in the pathogenesis and progression of GCA.

### 4.4. The Dual Roles of Senescence

A fundamental question in aging research is whether aging is entirely detrimental. Surprisingly, individuals with CHIP have been reported to have a lower risk of developing Alzheimer’s disease, a prototypical age-related disorder. Furthermore, among CHIP carriers who have not developed dementia, neuropathological changes—such as amyloid plaques and tau pathology—have been observed to be relatively mild [80,81]. One hypothesis proposes that myeloid cells bearing CHIP-associated mutations may infiltrate and engraft in the brain, potentially compensating for the age-related functional decline of resident microglia by acting as microglia-like cells [82].

In a murine model of liver fibrosis, the elimination of *p16 Ink4a*-positive macrophages (senescent macrophages) resulted in an improvement in fibrosis. In contrast, depletion of p16 Ink4a-positive endothelial cells (senescent endothelial cells) led to a worsening of fibrosis. These findings suggest that, while senescent macrophages exacerbate fibrosis, senescent endothelial cells may exert protective effects by expressing anti-fibrotic and reparative factors such as VEGF, thereby contributing to tissue repair and angiogenesis [83]. Thus, senescent cells are not uniformly detrimental, and future senolytic therapies will likely require cell-type-specific targeting [84].

## 5. Omics-Based Pathophysiological Insights

Recent advances in omics technologies have significantly enhanced our understanding of the molecular mechanisms underlying GCA. Through transcriptomic, proteomic, and spatial transcriptomic analyses, diverse immune pathways, cell types, and potential therapeutic targets have been identified. This section provides an overview of the key findings from recent omics-based studies, as summarized in Table 1, with particular emphasis on their implications for therapeutic intervention.

The role of CD4+ T cells has garnered significant attention as a key immune component in the pathophysiology of GCA. Single-cell RNA sequencing (scRNA-seq) analysis has revealed a marked expansion of cytotoxic CD4+ T cells (CTLs) expressing identical TCR sequences during active disease. These CTLs exhibit high expression levels of granzyme genes (*GZMB*, *GZMK*) and chemokine genes (*CCL4*, *CCL5*), suggesting their involvement in apoptosis induction, pro-inflammatory responses, and the recruitment of other immune cells. Notably, Maraviroc, a CCR5 antagonist that targets the receptor for *CCL5*, has been proposed as a potential therapeutic agent [85].

In relation to CD8+ T cells, an increased frequency of Ki-67-positive cells has been observed in the peripheral blood of patients with GCA, indicating a proliferative phenotype. These cells display a reduced activation threshold for TCR stimulation and can be activated even in the absence of co-stimulatory signals. Indeed, the scRNA-seq analysis of PBMCs demonstrated upregulation of genes such as *KLRD1* and *IFITM1*, which are associated with antiviral responses and immune activation, and downregulation of *GNLY* and *ZFP36L2*, which are involved in cytotoxic function and proliferative control. These gene expression profiles are consistent with previous findings. Furthermore, although CD8+ T cells in affected vascular lesions expressed IFN-γ, they lacked Ki-67 expression, suggesting that these cells were not undergoing local clonal expansion but were instead recruited from the peripheral circulation following prior activation and proliferation [86].

In studies investigating the histological heterogeneity of GCA, gene expression profiles have been compared between two subtypes of TAB tissue: the transmural inflammation (TMI) type and the inflammation-limited-to-the-adventitia (ILA) type. In the TMI subtype, a broad range of immune-activating molecules—including members of the TNF superfamily, immune checkpoints, chemokines and their receptors, TLRs, complement components, IgG Fc receptors, signaling lymphocytic activation molecule (SLAM) family members, *JAK3*, *STAT1*, and *STAT4*—were significantly upregulated. In contrast, the gene expression profile of the ILA subtype resembled that of normal tissue, indicating that TMI-type GCA exhibits a transcriptional profile reflective of marked inflammation. These findings highlight distinct molecular signatures that correspond to different histopathological classifications of GCA [87].

A spatial transcriptomics study further demonstrated upregulated gene expression across all arterial layers in TAB specimens from patients with GCA, with the most pronounced changes observed in the intima and media. In addition to increased expression of macrophage-related genes such as *MMP2* and *MMP9*, *CD74* was identified as the most highly expressed gene across all layers. *CD74*, a transmembrane receptor for macrophage migration inhibitory factor (MIF), is expressed on antigen-presenting cells and is known to be involved in apoptosis and tissue repair. These findings support the notion that angiogenic activity is primarily localized to the intima and media and suggest that several druggable molecular targets—including *CD74*, *MMPs*, and *CXCR4*—may represent promising candidates for future therapeutic development [88].

Furthermore, spatial transcriptomics combined with pseudotime analysis demonstrated a sustained increase in *MMP12* expression throughout the disease course, reaching up to a 50-fold elevation. These findings suggest that *MMP12* may serve as a potential biomarker reflecting disease activity in GCA [89]. In addition, trans-omics analysis of systemic vasculitis revealed that MMP12 is specifically expressed by CD206+ macrophages and multinucleated giant cells. Notably, MMP12 expression remained indicative of disease activity even under IL-6 blockade, suggesting its potential utility as a predictor of disease relapse [90].

## 6. Current Therapeutic Strategies and Their Limitations

**Figure 4:**
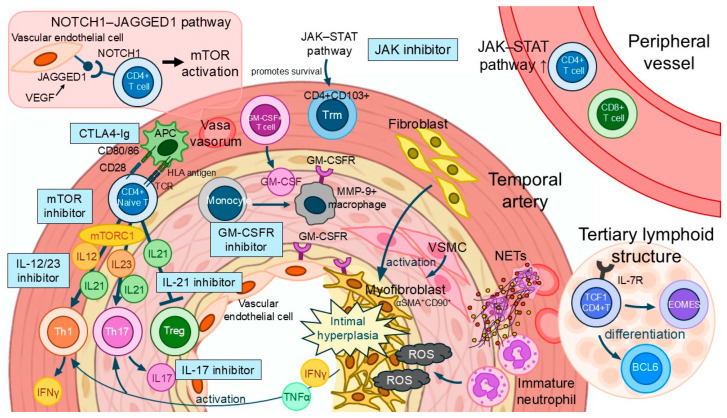
Cellular and molecular mechanisms underlying vascular inflammation in giant cell arteritis and potential therapeutic targets. This schematic illustrates the immunopathogenesis of GCA within the temporal artery, highlighting the spatial organization of immune cells and their interactions with vascular stromal components. CD4+ T cells become activated via antigen presentation by APCs in the adventitial vasa vasorum and differentiate into effector subsets, including Th1, Th17, Treg, and Trm. Key inflammatory pathways include the IL-12/23–Th1 axis, IL-6/IL-21–Th17 axis, and GM-CSF–macrophage axis, all of which contribute to vascular injury and intimal hyperplasia. Monocyte-derived macrophages express MMP-9 and interact with VSMCs and myofibroblasts, promoting structural remodeling and the production of ROS. The NOTCH1–JAGGED1–mTOR signaling cascade, as well as the JAK–STAT pathway, supports CD4+ T-cell survival and activation. Infiltrating neutrophils form NETs, further amplifying inflammation. Tertiary lymphoid structures containing TCF1+ CD4+ T cells, BCL6+ T follicular helper-like cells, and EOMES+ subsets are also observed. Multiple therapeutic targets are highlighted, including inhibitors of the mTOR, IL-12/23, IL-17, IL-21, GM-CSFR, CTLA-4, and JAK pathways. Abbreviations: APCs, antigen-presenting cells; BCL6, B-cell lymphoma6+ T follicular helper-like T cell; CTLA-4, cytotoxic T-lymphocyte antigen 4; EOMES, Eomesodermin+ cytotoxic T cell; GM-CSF, granulocyte–macrophage colony-stimulating factor; MMP, matrix metalloproteinase; NET, neutrophil extracellular trap; ROS, reactive oxygen species; Treg, Regulatory T cell; Trm, tissue-resident memory T cell; VSMC, vascular smooth muscle cell.

In this section, we provide an overview of the current therapeutic challenges and the therapeutic targets that have been proposed to date. The pathophysiology of GCA involves diverse immune cells and cytokine pathways, and understanding these mechanisms is essential for the development of novel therapeutic strategies. A conceptual diagram summarizing these pathogenic mechanisms is presented in Figure 4.

### 6.1. Glucocorticoid

Glucocorticoid (GC) remain the cornerstone of remission induction in GCA, exerting their anti-inflammatory effects through downregulation of pro-inflammatory genes, upregulation of GC receptor targets (e.g., FKBP5), and increased expression of anti-inflammatory markers such as CD163 [91,92].

However, in GCA patients treated with GC, a second TAB performed on the contralateral side revealed persistent vascular inflammation in approximately half of the patients, even one year after treatment initiation [93]. Moreover, prolonged GC use is associated with considerable toxicity. A recent study comparing patients receiving methylprednisolone pulse therapy followed by oral GC with those receiving GC alone demonstrated no significant difference in visual recovery, while the risk of developing diabetes within one year was significantly higher in the pulse group [94]. Furthermore, reports have indicated that even a 26-week rapid GC tapering regimen does not adequately prevent GC-related adverse events [95]. These adverse effects warrant particular attention in elderly patients with GCA.

### 6.2. Tocilizumab

The addition of tocilizumab (TCZ), an IL-6 inhibitor, significantly reduces relapse rates and cumulative GC exposure in GCA, establishing it as a major therapeutic option [96]. In real-world clinical practice in Japan, 85% of patients were able to taper GC without relapse at 52 weeks [97]. Furthermore, TCZ has been reported to prevent new visual complications and may reduce the risk of blindness [98].

However, tapering or discontinuation of TCZ is associated with relapse risk [99], and approximately 47% of patients with GCA relapse within five years [100]. In a study of patients with GCA treated with GC and TCZ who underwent re-biopsy at a median of 2.4 years after treatment initiation, persistent vasculitis was observed in about 40% of cases, despite well-controlled clinical disease activity [101]. These challenges highlight the need for more fundamental therapeutic strategies.

### 6.3. JAK Inhibitor

JAK inhibitors target intracellular signaling downstream of multiple pro-inflammatory cytokines (IL-6, IFN-γ, GM-CSF). Baricitinib, tofacitinib, and upadacitinib have been demonstrated to suppress vascular inflammation in murine models and to achieve clinical remission in approximately 60% of patients with relapsing GCA in real-world settings, including those refractory to IL-6 blockade [102]. In a Phase 3 trial of upadacitinib (a JAK1-selective inhibitor) in GCA, sustained remission rates at 52 weeks were significantly higher (15 mg (46%) vs. placebo (29%)), with additional steroid-sparing effects [103]. Based on these results, upadacitinib has been approved for the treatment of GCA. However, the potential cardiovascular and malignancy risks associated with JAK inhibitors require careful consideration [104].

### 6.4. GM-CSFR Inhibitor

GM-CSF and its receptor (GM-CSFRα) are upregulated in GCA lesions, particularly in macrophages, T cells, and vascular endothelial cells, where they activate JAK2/STAT5 signaling. Mavrilimumab, a monoclonal antibody targeting GM-CSFRα, has been shown in ex vivo cultures of GCA arteries to reduce inflammatory infiltrates, pro-inflammatory cytokines (IL-6, TNFα, IL-1β), oxidative stress, and angiogenesis [105]. In a Phase 2 trial, mavrilimumab demonstrated superior efficacy to placebo in achieving sustained remission, yielding promising results; however, a Phase 3 trial is not currently underway [106].

### 6.5. CTLA-4 Ig

As noted above, dysregulation of immune checkpoint pathways contributes to the pathophysiology of GCA. Abatacept (CTLA-4 Ig), which modulates T-cell co-stimulation via CD80/86, demonstrated superiority over standard therapy in achieving relapse-free survival in a Phase 2 trial of GCA [107]. A Phase 3 trial is currently underway (NCT04474847).

### 6.6. IL-17 Inhibitor

IL-17A is highly expressed in GCA arteries and correlates with the intensity of inflammation. Secukinumab (a monoclonal antibody against IL-17A) showed promising results in a Phase 2 trial (70% remission maintenance vs. 20% with placebo at 28 weeks). Phase 3 trials are currently ongoing (NCT04930094, NCT05380453) [108].

### 6.7. IL-12/IL-23 Inhibitor

IL-23 is a key cytokine for the proliferation and maintenance of Th17 cells. Inflammatory aortic aneurysms exhibit upregulated expression of the IL-23 receptor gene, suggesting an association with GCA [109]. However, studies of Ustekinumab (an IL-12/23 inhibitor) in GCA have not demonstrated consistent efficacy. Furthermore, a clinical trial of Guselkumab (an anti–IL-23 antibody) was terminated (NCT04633447) [108]. Thus, although targeting the IL-12/23 pathway remains theoretically promising, its clinical efficacy in GCA has yet to be established.

### 6.8. IL-21 Inhibitor

IL-21 is primarily produced by Tfh cells and has been reported to promote B-cell activation and support the maintenance of effector CD8+ T-cell responses [110]. Its expression is epigenetically suppressed by the transcription factor Blimp-1, whose downregulation is frequently observed in autoimmune diseases. Inhibition of IL-21 has been shown to decrease Th1 and Th17 cells, while potentially increasing Th2 cells with protective functions [111]. However, evidence for its clinical application in GCA remains limited.

## 7. Emerging Immune Cells and Potential Therapeutic Targets

In this section, we provide an overview of the major immune cell populations that have recently gained attention in the pathogenesis of GCA and examine their potential as therapeutic targets.

### 7.1. Macrophage

Macrophages are the primary cellular source of IL-6, MMP-9, and VEGF, and they play a pivotal role in mediating vascular wall destruction and remodeling in GCA [112,113]. The ontogeny of macrophages varies depending on the inflammatory state and their localization within the arterial wall [114].

Fate-mapping analyses combined with single-cell RNA sequencing have revealed that the developmental origin of arterial macrophages differs between homeostatic and inflammatory conditions. Under steady-state conditions, arterial macrophages are derived equally from yolk sac erythro-myeloid progenitors and the bone marrow, whereas during vascular inflammation, the majority originate from bone marrow-derived circulating monocytes that differentiate into adventitial macrophages. Notably, a subset of adventitial macrophages, characterized by *LYVE1* expression and derived from the bone marrow under homeostatic conditions, may play a reparative role that appears to decline with age [115].

In a study investigating the pathogenesis of intimal inflammation using coronary artery specimens from patients with Kawasaki disease, intimal macrophages were found to express the chemokine receptor *CCR2*, suggesting their derivation from circulating monocytes. These macrophages are believed to contribute to vascular occlusion, remodeling, and thrombus formation [116].

Moreover, macrophages in GCA exhibit phenotypic diversity and functional heterogeneity, with distinct spatial distribution patterns depending on the disease stage. CD206+/MMP-9+ macrophages, which are involved in tissue destruction and angiogenesis, are induced in the early phase by GM-CSF and are localized to areas of tissue damage. In contrast, FRβ+ macrophages are induced in the later stages by macrophage colony-stimulating factor and are hypothesized to activate myofibroblasts and promote intimal hyperplasia, thereby contributing to luminal stenosis [114,117].

Recent transcriptomic analyses of treatment-naïve GCA lesions have identified macrophages and multinucleated giant cells expressing a wide array of molecules implicated in GCA pathogenesis, including MMP12 (critical for elastin degradation), *ACP5* and *ATP6V0D2* (osteoclast-associated bone resorption molecules), *VDR* and *TREM2* (involved in osteoclastogenesis and phagocytosis), *MRC1* (a marker of fibrosis-associated macrophages), and *HLA-DRA* (involved in antigen presentation to CD4+ T cells) [118]. These molecules are not unique to GCA but are also expressed in other granulomatous diseases such as sarcoidosis, tuberculosis, and TAK, suggesting the potential for shared therapeutic targets across these conditions.

### 7.2. Tissue-Resident Memory T Cell (Trm)

In patients with GCA definitively diagnosed by TAB, a study that performed a second TAB on the contralateral side revealed that approximately half of the patients exhibited persistent vascular inflammation even one year after the initiation of treatment [93]. Histopathological analysis demonstrated that T cells were the predominant residual cell population, drawing increasing attention to tissue-resident memory T cells (Trm) [68]. Trm are a subset of memory T cells that persist long term within tissues without recirculating. Upon reactivation, they exert local effector functions, including Th1-, Th2-, and Th17-type responses.

In a murine model of GCA, CD4+ CD103+ Trm were found to reside within the arterial wall [119]. In this study, arteries containing Trm were transplanted into SCID mice without the addition of PBMCs, yet the Trm remained viable over the long term and continued to produce cytokines such as IFN-γ, IL-17, and IL-21. The survival, maintenance, and cytokine production of Trm were shown to depend on CD28-mediated co-stimulation, which enhances glucose uptake in T cells, and on JAK1/3-STAT signaling for sustained activity [120]. Indeed, it has been suggested that tofacitinib may have therapeutic efficacy against Trm [119,121].

Recent omics analyses have identified *DDIT4* and *ARHGAP15* as susceptibility genes associated with GCA. In particular, *DDIT4* promotes mitochondrial fission and contributes to the maintenance of chronic inflammation in Trm by regulating lipid metabolism and reactive oxygen species (ROS). Notably, deletion of *DDIT4* resulted in a significant reduction in chronic inflammation, highlighting its potential as a novel therapeutic target [122].

### 7.3. Stem-like T Cell

Recent studies have reported the formation of lymph node-like tertiary lymphoid structures within the aortic wall of patients with GCA, where clusters of CD4+ T cells and B cells are present. Among these, TCF1 hi IL-7R+ stem-like CD4+ T cells express the IL-7R and exhibit stem cell-like properties, including self-renewal and the ability to differentiate into Eomesodermin (EOMES)+ cytotoxic T cells and B-cell lymphoma 6+ follicular helper-like T cells. These stem-like CD4+ T cells play a central role in sustaining chronic inflammation. Notably, in mouse models, the depletion of these cells resulted in the prevention of vasculitis relapse [123].

EOMES is a transcription factor essential for the differentiation of T cells—particularly CD8+ T cells—into memory cells and for their cytotoxic function. T cells harboring *TET2* mutations exhibit increased EOMES expression and display a bias toward differentiation into memory CD8+ T cells. As a result, although primary immune responses may remain intact, secondary responses—such as those elicited upon reinfection—may become exaggerated [32]. Eliminating IL-7R+ TCF1hi CD4+ stem-like T cells in TLS or inhibiting TLS formation represents a novel strategy to prevent relapse [123].

### 7.4. B Cell

Although the pathogenesis of GCA has traditionally been investigated primarily through the lens of cellular immunity, recent studies have increasingly emphasized the role of humoral immunity. In GCA, B cells that infiltrate the vessel wall from the adventitial vasculature form tertiary lymphoid organs and secrete a range of inflammatory cytokines—including IL-6, GM-CSF, TNF-α, and lymphotoxin-β (LTβ)—as well as the anti-inflammatory cytokine IL-10. These B cells may promote the differentiation of macrophages migrating from the vasculature toward the media into a proinflammatory phenotype [124].

Moreover, a recent study reported that 57% of GCA patients tested positive for anti-*VSIG10L* antibodies and 43% for anti-*DCBLD1* antibodies. In contrast, these antibodies were not detected in patients with TAK or in healthy controls, suggesting that antibody-mediated humoral immunity may also play a role in the pathogenesis of GCA [125]. These findings highlight the potential of B cell-targeted therapies.

### 7.5. Vascular Smooth Muscle Cell

Vascular smooth muscle cells (VSMCs) play a pivotal role in vascular inflammation and remodeling. In vitro, they respond to stimulation with IFN-γ or PDGF by producing chemokines such as CXCL9, CXCL10, CXCL11, and CCL2, thereby promoting the chemotaxis of monocytes and Th1 cells and recruiting CD8^+^ T cells into the vessel wall. In addition, VSMCs release matrix metalloproteinases (MMP-2 and MMP-9) and interact with fibroblasts and myofibroblasts to drive intimal hyperplasia and vascular occlusion [6]. Experimental studies have further demonstrated that inhibition of PDGF signaling with imatinib reduces VSMC-derived CCL2 production, thereby attenuating T-cell chemotaxis [126].

### 7.6. Myofibroblast

Myofibroblasts contribute to intimal hyperplasia and vascular occlusion. Their origins vary depending on the organ and tissue context, arising from multiple cell types [127]. In GCA, myofibroblasts are believed to differentiate from various sources, including vascular smooth muscle cells [128,129], endothelial cells [17], and fibroblasts [130]. Morphologically, myofibroblasts are irregular, spindle-shaped cells with high migratory and proliferative capacities, and they exhibit approximately twice the contractile strength of quiescent fibroblasts [127]. Myofibroblasts produce profibrotic mediators such as TGF-β1, angiotensin II, and IL-1, thereby contributing to both fibrosis and tissue repair [131].

Recent studies have suggested that, in temporal artery specimens from patients with GCA, adventitial CD90+ fibroblasts differentiate into activated fibroblasts that migrate from the adventitia toward the intima, contributing to neointimal formation [132]. Signals that induce fibroblast-to-myofibroblast differentiation include TGF-β signaling and IL-6 [131].

Neointimal myofibroblasts (characterized by α-SMA+ CD90+ Desmin+ MYH11+ expression) in GCA lesions are not merely structural cells but are immunologically active. These cells exhibit activated IFN-γ signaling, as evidenced by phosphorylated STAT1, and promote Th1/Tc1 and Th17/Tc17 polarization by producing IL-12 and IL-23, thereby creating a pro-inflammatory microenvironment. Accordingly, JAK inhibitors represent a potential therapeutic strategy [6,133]. Furthermore, recent genome-wide gene expression profiling has revealed that myofibroblasts involved in intimal hyperplasia express *LRRC15*, suggesting that these cells contribute to the formation of an immunosuppressive microenvironment by inhibiting cytotoxic CD8+ T cells and highlighting *LRRC15* as a potential therapeutic target [118].

### 7.7. Neutrophil

In chronic inflammatory states, immature neutrophils are released into peripheral blood, and patients with GCA exhibit significantly higher numbers of circulating immature neutrophils compared to healthy individuals. These immature neutrophils have a longer lifespan than their mature counterparts and demonstrate resistance to apoptosis. They produce large quantities of ROS, disrupt the vascular endothelial barrier, and increase vascular permeability [134,135].

Temporal artery biopsies from GCA patients have revealed the presence of NETs, primarily localized near the vasa vasorum in the adventitia. These NETs contain proinflammatory cytokines such as IL-6 and IL-17A [136]. Mitochondrial-derived N-formyl methionine peptides (*fMET*), released during NET formation, act as potent neutrophil chemoattractants and correlate strongly with inflammatory markers such as C-reactive protein (CRP) and erythrocyte sedimentation rate. *fMET* induces ROS production via the G-protein-coupled receptor formyl peptide receptor 1 (*FPR1*), and inhibition of *FPR1* suppresses ROS generation [137]. Moreover, ROS production is elevated in GCA leukocytes, contributing to oxidative modifications of fibrinogen and potentially promoting thrombosis. This effect is attenuated by treatment with tocilizumab [138].

These potential therapeutic targets remain under investigation, and further studies are needed to evaluate their efficacy and facilitate their translation into clinical practice.

## 8. Conclusions and Future Perspectives

Recent studies have revealed that age-related mechanisms, including immunosenescence and CHIP, contribute to the pathogenesis of giant cell arteritis. Moreover, emerging evidence has highlighted the roles of macrophages, Trm, and stromal cells as novel immune players in disease progression. Integrating these insights with multi-omics approaches holds promise for developing precision medicine strategies aimed at achieving sustained remission and preventing irreversible vascular damage.

## Figures and Tables

**Table 1:** Summary of recent omics-based studies investigating the pathogenesis of giant cell arteritis. This table provides a summary of recent studies employing transcriptomic, proteomic, and spatial omics approaches to investigate the immunopathology of GCA. The columns present the year of publication, type and source of specimens (e.g., peripheral blood mononuclear cells [PBMCs], temporal artery biopsy [TAB], arterial tissue), sample sizes (N = number of cases; C = number of controls), analytical platforms used, key findings, and potential therapeutic targets. The final column provides PubMed IDs (PMIDs) for reference. * Publicly available datasets were used exclusively. Analysis was performed by integrating data from GSE157007 (PMID: 37117750) and GSE198891 (PMID: 35460236). Abbreviations: scRNA-seq, single-cell RNA sequencing; SV, systemic vasculitis; TCR, T-cell receptor; TLS, tertiary lymphoid structure; TMI, transmural inflammation; IL, interleukin; ROS, reactive oxygen species.

Year	Specimen	N	C	Modality/Platform	Key Findings/Potential Therapeutic Target	PMID
2022	GCA PBMC	3	3	scRNA-seq /10x Chromium	Genes associated with antiviral defense and immune activation (*KLRD1*, *IFITM1*) were upregulated, whereas those involved in cytotoxicity and proliferation control (*GNLY*, *ZFP36L2*) were downregulated.	35460236
2022	GCA Arterial tissue	3	None	scRNA-seq, TCR repertoire /10x Chromium	TCF1+IL7R+ stem-like CD4+ T cells expand clonally in TLSs, suggesting IL-7R+ CD4+ T cells as pathogenic and therapeutic targets.	37672564
2023	GCA TAB	9	7	Spatial transcriptomics /NanoString GeoMx DSP	CD74 and macrophage-associated pathways—including *MMP2*, *MMP9*, and *CXCR4*—were identified as key upregulated targets in GCA, particularly within the intima and media layers.	37744332
2024	GCA PBMC	8	8	scRNA-seq, TCR repertoire /10x Genomics Chromium	Clonally expanded cytotoxic CD4+ T cells in active GCA highly expressed *GZMB* and *CCL5*, suggesting Maraviroc as a potential therapy.	37952293
2024	GCA TAB	49	7	Gene expression profiling /NanoString nCounter	In TMI lesions, genes encoding TNF superfamily members, immune checkpoints, chemokines and their receptors, toll-like receptors, complement components, Fc receptors for IgG, signaling lymphocytic activation molecules, as well as *JAK3*, *STAT1*, and *STAT4* were upregulated.	39317454
2024	GCA PBMC *	(a) 40 (b) 14	(a) 31 (b) 6	(a) Bulk RNA-seq (b) scRNA-seq /Illumina, 10x Chromium	Targeting *DDIT4*, a causal gene for GCA, may suppress persistent inflammation in CD4+ memory T cells.	39762453
2024	GCA TAB	10	6	Gene expression profiling /Agilent microarray	*MMP12*, *ACP5*, and *TREM2* are potential therapeutic targets in GCA, associated with macrophage-driven tissue destruction, while *LRRC15* marks myofibroblasts contributing to intimal hyperplasia in granulomatous inflammation.	39837478
2025	SV (c) Serum, whole blood, tissue	332 (c) GCA2, GPA1	30	Proteome analysis, RNA sequencing, Spatial transcriptomics /Olink Proteomics, Illumina. 10x Genomics	*MMP12* is selectively elevated in vasculitis and specifically expressed in CD206-positive macrophages and multinucleated giant cells within lesions. It reflects disease activity even under IL-6 blockade and helps predict relapse.	40139687

## Data Availability

Not applicable.
